# Facial nerve paralysis in 64 equids: Clinical variables, diagnosis, and outcome

**DOI:** 10.1111/jvim.15767

**Published:** 2020-04-06

**Authors:** Sophie Boorman, Nicole M. Scherrer, Darko Stefanovski, Amy L. Johnson

**Affiliations:** ^1^ Department of Clinical Sciences J. T. Vaughan Teaching Hospital, Auburn University College of Veterinary Medicine Auburn Alabama USA; ^2^ Department of Clinical Studies, New Bolton Center University of Pennsylvania Kennett Square Pennsylvania USA

**Keywords:** cranial nerve, equine, neurological deficits, ophthalmology, peripheral neuropathy

## Abstract

**Background:**

Facial nerve paralysis (FNP) in equids is not well described in the veterinary literature.

**Objective:**

To investigate the causes of FNP and associations among clinical variables, diagnosis, and outcome.

**Animals:**

Sixty‐four equids presenting with FNP between July 2000 and April 2019. Cases of postanesthetic FNP were excluded.

**Methods:**

Medical records were retrospectively reviewed. Variables were evaluated for associations with outcomes (diagnosis and case outcome) using logistic regression.

**Results:**

The most common cause of FNP was trauma (n = 20). Additional diagnoses included central nervous system (CNS) disease (n = 16), idiopathic (n = 12, 4 of which had adequate diagnostic investigation and were considered “true” idiopathic, and 8 of which were considered “not investigated” idiopathic), temporohyoid osteoarthropathy (n = 10), otitis media‐interna (n = 3), lymphoma (n = 1), iatrogenic as a consequence of infiltration of local anesthetic (n = 1), and clostridial myositis (n = 1). Follow‐up was available for 55 (86%) cases. Twenty‐nine (53%) equids had full resolution of FNP, 14 (25%) were euthanized, 6 (11%) partially improved, and 6 (11%) were unchanged or worse.

**Conclusions and Clinical Importance:**

If FNP is the consequence of CNS disease, successful treatment of the primary disease likely leads to resolution of FNP. Most cases of FNP in equids are traumatic in origin. True idiopathic cases are uncommon.

**Abbreviations**
ACVIM: American College of Veterinary Internal Medicine
CI: confidence interval
CNS: central nervous system
CSF: cerebrospinal fluid
CT: computed tomography
DMSO: dimethyl sulfoxide
EEE: eastern equine encephalitis
EHV‐1: equine herpes virus‐1
EPM: equine protozoal myeloencephalitis
IQR: interquartile range
MMS: modified Mayhew scale
MRI: magnetic resonance imaging
OR: odds ratio
Osp: outer surface protein
SAG 2,3,4: surface antigens 2,3,4
THO: temporohyoid osteoarthropathy
UAE: upper airway endoscopy
WNV: West Nile virus

## 1 INTRODUCTION

Facial nerve paralysis (FNP) manifests frequently as a unilateral dysfunction of the muscles of facial expression.^1^ In equids, FNP results in muzzle deviation, ptosis, partial or complete inability to blink, and abnormal ear position. Consequences of FNP include decreased cosmesis, dropping feed while chewing (“quidding”), ptyalism because of loss of lip muscle tone, and corneal ulceration, all of which prompt owners to seek veterinary evaluation.

In contrast to equids, the potential causes of FNP are well described in small animals and in people. Described causes of FNP in dogs include otitis media‐interna, trauma, hypothyroidism, diabetes mellitus, neoplasia, brainstem lesions, listeriosis, myasthenia gravis, botulism, and sulfonamide hypersensitivity.^2^ The most common classification, however, is idiopathic.^3^ Likewise, in humans, FNP can result from many causes. Important causes of FNP include meningitis, multiple sclerosis, human immunodeficiency virus, stroke, facial nerve tumors, otitis media, and Lyme disease.^4^ Similar to dogs, however, 70% of cases are idiopathic, a condition known as Bell's palsy.^4^ This condition is not uncommon, with 11 to 40 cases per 100 000 people and typically it has a rapid onset, is unilateral, and is self‐limiting.^4^ However, Bell's palsy can become chronic.^5^ To our knowledge, no published reviews of causes of FNP in the horse are available.

The known causes of FNP in horses include central nervous system (CNS) diseases affecting the brainstem, such as equine protozoal myeloencephalitis (EPM) or neuroborreliosis, diseases of the tympanic cavity or guttural pouch, such as temporohyoid osteoarthropathy (THO), and trauma, most commonly to the petrous temporal bone or peripheral branches of the facial nerve. Reports also exist of FNP caused by hypothyroidism and congenital malformation of the facial nerve.^6^, ^7^ Because idiopathic FNP is common in small animal and human medicine, it would reasonably follow that idiopathic FNP might also be common in equids. Thus far no published reports of FNP in horses describe cases for which thorough investigation has been conducted and no specific diagnosis reached. This absence might reflect lack of consensus on how to diagnose idiopathic FNP or whether such a diagnosis truly exists in equids.

As well as limited information on the most common causes of FNP in the horse being available, little is known about the prognosis. Different causes of FNP likely result in different amounts of neuronal damage and thus may have different prognoses. Also, little information about associations among clinical variables and the known causes of FNP is available for equids.

The first aim of our retrospective study was to investigate the causes of FNP in the horse and to report statistical associations between clinical variables and diagnosis. Our second aim was to determine the prognosis for recovery of facial nerve function by describing associations among clinical variables, diagnosis, treatments, and outcomes.

## 2 MATERIALS AND METHODS

Medical records of equids presenting to the University of Pennsylvania's George D. Widener Hospital for Large Animals at New Bolton Center between July 2000 and April 2019 with FNP were reviewed. Equids that developed FNP as an anesthetic complication during hospitalization were excluded. Such cases are underreported in our hospital medical record system and therefore are impossible to accurately quantify. The underreporting is likely because postanesthetic FNP often is a mild and self‐limiting complication that resolves before hospital discharge, resulting in failure to record FNP as a diagnosis.

### 2.1 Demographic and pertinent history

Demographic data gathered included age, breed, and sex. Historical information included duration of FNP, presenting complaint, history of ocular pathology, and trauma. Duration of FNP was classified as either acute (developed within 7 days) or chronic (present 8 days or longer).

### 2.2 Clinical presentation

Physical examination findings were noted, including rectal temperature, respiratory rate, heart rate, and ophthalmological examination findings. The presence or absence of the following clinical signs of FNP was recorded: ptosis, ear droop, muzzle deviation, absent blink, and lip droop. Facial nerve paralysis was further classified as bilateral or unilateral; for unilateral cases, laterality was noted. Other clinical signs, including neurological examination findings, were recorded.

### 2.3 Diagnostic investigation

If a neurological gait examination was performed by an American College of Veterinary Internal Medicine (ACVIM) diplomate, modified Mayhew score (MMS) for ataxia was recorded.^8^ Clinicopathological results were recorded, including hematology, serum biochemistry, plasma fibrinogen concentration, and blood lactate concentration. Results were compared to reference ranges for horses.^9^ For statistical analysis, if ≥5 cases had a common clinicopathological abnormality (eg, hyperglycemia), all cases were recorded as having the abnormality either present or absent. Cerebrospinal fluid (CSF) analysis results were noted if performed, including antibody test results for *Sarcocystis neurona* and *Borrelia burgdorferi*. If upper airway endoscopy (UAE) including guttural pouch examination was performed, results were described and classified as normal or abnormal. Diagnostic imaging results were recorded and classified as normal or abnormal. If additional diagnostic tests were performed, results were recorded.

### 2.4 Diagnosis, treatments, and outcomes

Final diagnosis was recorded and categorized as “trauma,” “THO,” “CNS diseases” (subcategorized as “EPM,” “neuroborreliosis,” “idiopathic,” “other”) and “other.” Equids with a diagnosis of trauma required either a history of trauma, clinical signs of trauma, or evidence of trauma on diagnostic imaging (eg, skull fracture). Equids with endoscopic or radiographic evidence of proliferative change to the temporohyoid joint and stylohyoid bone were diagnosed with THO.^10^ To be considered positive for EPM, a *S. neurona* serum : CSF antibody titer ratio of <100 was required.^11^ Patients were given a tentative antemortem diagnosis of neuroborreliosis if they had a serum : CSF ratio of <130 for antibodies to *B. burgdorferi* outer surface proteins, and all other likely diseases including EPM were excluded by appropriate diagnostic testing.^12^, ^13^ Equids that were categorized as having idiopathic FNP were further grouped as “true idiopathic” or “not investigated”; “true idiopathic” was defined as cases that had no history or clinical signs of trauma, normal CSF analysis, normal UAE, and normal diagnostic imaging of the skull. “Not investigated” was defined as no history or clinical signs of trauma and no cause identified for FNP but without utilization of all 3 diagnostic modalities (CSF analysis, guttural pouch endoscopy, diagnostic imaging of the skull). Treatment provided was documented, including surgical and medical treatment (eg, antimicrobials, analgesics, ophthalmological treatments). Total number of days hospitalized was noted. Outcome was obtained either from medical records or from owners by telephone and was classified as “euthanized,” “FNP fully resolved,” “FNP partially improved,” or “FNP unchanged or worse.”

### 2.5 Statistical analysis

All analyses were conducted using Stata 15MP (StataCorp, College Station, Texas). A *P* value <.05 was considered significant. Descriptive statistics were reported either as frequency counts (percentages of total) for categorical variables or as medians and interquartile range (IQR; lower quartile, upper quartile) for continuous variables. Univariate logistic regression was used to identify statistically significant variables (*P* < .05) associated with the clinical diagnoses and case outcome. A complete list of the variables analyzed is provided in Data S1.

## 3 RESULTS

### 3.1 Demographic

Sixty‐four equids (63 horses and 1 donkey) were identified with FNP. The animals were between 2 months and 25 years of age (median, 8 years; IQR, 4‐16 years). Seven different breeds of horse were represented, including 31 Thoroughbreds, 10 Warmbloods, 8 Quarter Horses, 6 Standardbreds, 3 Draft breeds, 3 Arabians, and 2 ponies. There were 30 castrated males, 29 mares, and 5 intact males.

### 3.2 Pertinent history

For the purpose of statistical analysis, presenting complaints were categorized as “FNP” in 30% (19/64) of cases, “trauma” in 23% (15/64) of cases, “other neurological problem” in 23% (15/64) of cases, “generalized weakness or malaise” in 14% (9/64) of cases and “other” in 9% (6/64) of cases. See Data S1 for a complete list of presenting complaints. Five equids had a history of a nonhealing corneal ulcer and were presented after having begun ophthalmic treatment. Duration of FNP was known in 48 cases and ranged from 2 hours to 1 year (median, 3 days; IQR, 1‐14.75 days). Facial nerve paralysis was considered acute in 63% (30/48) of cases.

### 3.3 Clinical presentation

Facial nerve paralysis was unilateral in 92% (59/64) of cases and bilateral in 8% (5/64) of cases. Of those with unilateral FNP, 54% (32/59) had right‐sided disease and 46% had left‐sided (27/59) disease. Sixty‐nine percent (44/64) of cases had deviation of the muzzle away from the affected side, and 63% (40/64) had drooping of the lip. An ear droop or bilateral ear droop was present in 55% (35/64) of cases. Inability to blink the eye on the affected side was recorded in 53% (34/64) of cases and ptosis of the eye on the affected side was present in 52% (33/64) of cases. Fifty‐three percent (34/64) of cases had ≥3 signs of FNP, and 16% (10/64) of cases had all 5 signs (ptosis, ear droop, muzzle deviation, absent blink, and lip droop). Additional signs attributed to FNP included nares collapse (3 cases, in 1 case bilateral, causing respiratory distress).

Vital parameter results were available in 81% (52/64) of cases. Median rectal temperature was 38.0°C (IQR, 37.2‐38.3); fever (≥38.6°C) was identified in 5 cases. Median heart rate was 44 beats per minute (IQR, 39‐53 beats per minute); tachycardia (≥45 beats per minute) was recorded in 30% (19/52) of cases; and median respiratory rate was 20 breaths per minute (IQR, 14‐24 breaths per minute).^14^

Ocular pathology on the affected side was noted in 36% (23/64) of cases, and some equids had multiple ophthalmic problems. One third (21/64) of the cases had corneal ulceration, 3 with absent or decreased pupillary light reflex, 1 with a decreased Schirmer tear test result, 1 with an upper eyelid laceration, and 1 with both uveitis and a cataract. Of the 5 animals with bilateral FNP, 3 had corneal ulcers: 2 had bilateral corneal ulcers and 1 had a single corneal ulcer.

Several other clinical signs not specific for FNP were noted at presentation and were broadly divided into neurological and nonneurological signs. Of the 55% (35/64) of animals that presented with neurological clinical signs: 60% (21/35) were ataxic, 34% (12/35) had head tilt, 29% (10/35) had change in mentation, 26% (9/35) had dysphagia or weak tongue, 17% (6/35) had fasciculations of the lips, tongue, or facial muscles, 3 had ventral strabismus, 2 had signs of seizures or collapse, and 2 had periods of recumbency or inability to rise. Neurological gait examination was performed by an ACVIM diplomate in 55% (35/64) of animals. Ataxia was noted in 60% (21/35) of these animals. The MMS was 1/5 (IQR, 0‐2) and scores ranged from 0 to 5.

Nonneurological clinical signs included lameness (9% [6/64] including 1 case of laminitis and 2 cases of septic arthritis), laceration or abrasions (9% [6/64] including 1 open orbital fracture), poor condition (4), SC emphysema of the head (2), masseter muscle atrophy (2), icterus (1), mucous membrane color suggestive of sepsis (1), heart murmur (1), colic (1), epistaxis (1), purulent aural discharge (1), diarrhea (1), alopecia over the ear (1), facial swelling (not caused by SC emphysema, 1), and head shyness (1).

### 3.4 Diagnostic investigation

#### 3.4.1 Clinicopathological results

Hematological analyses performed included PCV and total plasma proteins (66% of cases, 42/64), plasma fibrinogen concentration (66%, 42/64), CBC (63%, 40/64), and serum biochemistry (41%, 26/64). A summary of the results is presented in Data S1. An increased plasma fibrinogen concentration was noted in 57% (24/42) of cases (median, 399.5 mg/dL; range, 198‐691 mg/dL). Other common abnormalities included increased serum total bilirubin concentration (67% [12/18] of cases; median, 3.65 mg/dL; range, 0.8‐10.2 mg/dL) and hyperglycemia (46% [13/28] of cases; median, 123.9 mg/dL; range, 94.6‐178 mg/dL).

Cerebrospinal fluid analysis was obtained in 33% (21/64) of cases. Analysis of CSF nucleated cell count, red blood cell count, and protein concentration was performed in 90% (19/21) of cases; results are presented in Data S1. Cerebrospinal fluid cytology was performed in 90% (19/21) of these cases and was normal in 95% (18/19); in 1 case, cytology was reported as “neutrophilic inflammation.”

Immunodiagnostic testing for *S. neurona* utilizing either the combined surface antigen (SAG) 2, 4/3 ELISA or the indirect fluorescent antibody test was performed in 34% (22/64) of cases; 21 equids had both serum and CSF testing performed whereas 1 horse only had serum testing performed. The single case in which serum alone was tested was considered negative for EPM. In total, 16% (10/64) of cases were considered positive for *S. neurona* intrathecal antibody production.

Immunodiagnostic testing of serum and CSF for the presence of antibodies to *B. burgdorferi* outer surface proteins was performed in 20% (13/64) of cases and findings are summarized in Table 1.

**Table 1:** Lyme disease multiplex results (n = 13) in equids presenting with facial nerve paralysis

	Osp	Positive result (MFI cutoff)	Median (range) MFI	Number (%) with positive results
CSF	OspA	Unestablished	379 (54‐13 028)	N/A
	OspC	Unestablished	142 (18‐4502)	N/A
	OspF	Unestablished	599 (88‐17 468)	N/A
Serum	OspA	>2000	331 (76‐5772)	1 (8%)
	OspC	>1000	137 (30‐962)	0 (0%)
	OspF	>1250	585 (100‐4052)	3 (23%)

Abbreviations: CSF, cerebrospinal fluid; MFI, median fluorescent intensities; MRI, magnetic resonance imaging; Osp, outer surface proteins.

#### 3.4.2 Resting Upper Airway Endoscopy (UAE)

Examination of the upper respiratory tract including both guttural pouches was performed in 56% (36/64) of cases. Results were considered abnormal in 39% (14/36) of cases. Abnormal findings included thickening of 1 or both stylohyoid bones (10), healing stylohyoid fracture (1), grade 4 left laryngeal hemiplegia (1), persistently entrapped epiglottis (1), and inability to swallow (1). Sixty‐one percent (22/36) of equids had no clinically relevant findings. Results were recorded as no abnormal findings (16), slight irregularity of the stylohyoid bone (2), grade 1 left laryngeal hemiplegia (2), signs of resolved guttural pouch disease (1), and fasciculations of the arytenoid cartilages (1).

#### 3.4.3 Diagnostic imaging

Diagnostic imaging examinations included radiography of the skull (45%, 29/64), computed tomographic (CT) examination of the skull (8%, 5/64), ultrasonography of the dorsal and ventral buccal nerve branches (1), ultrasonography of the temporomandibular joint (1), full body nuclear scintigraphic examination (1), and magnetic resonance imaging (MRI) of the brain (1). Diagnostic imaging findings were considered abnormal in 17 equids. Abnormalities included skull fracture (7), thickening or proliferation of 1 or both stylohyoid bones (6), soft‐tissue or fluid density radiopacity in the tympanic bulla (2), osteolysis around a tooth root (1), increased radiopharmaceutical uptake in the frontal bone (1), and stylohyoid bone fracture (1).

#### 3.4.4 Additional diagnostic tests

Corneal cytology was performed in 5 cases. Other diagnostic tests included West Nile virus (WNV) and eastern equine encephalitis (EEE), IgM capture ELISA (2), equine herpes virus‐1 (EHV‐1) PCR (2), muscle biopsy (2), urinalysis (2), Schirmer tear test (2), culture and sensitivity of an abscess (1), otoscopy (1), and cytology of otic discharge (1). In most cases, results of these ancillary diagnostic tests were negative or did not influence the diagnosis. One horse, however, was positive for WNV.

### 3.5 Diagnosis

The most common cause of FNP was trauma, diagnosed in 31% (20/64) of cases. The FNP was caused by CNS disease in 25% (16/64) equids, including EPM in 16% (10/64) of patients, neuroborreliosis in 8% (5/64), and WNV in 1 horse. Nineteen percent (12/64) of cases were considered idiopathic. Of these, 4 were considered “true idiopathic” and 8 were “not investigated” (Table 2). Sixteen percent (10/64) of equids were diagnosed with THO. Other diagnoses included otitis media and externa (3), lymphoma (1), iatrogenic (caused by infiltration of local anesthetic to facilitate placement of a facial arterial catheter) (1), and clostridial myositis (1).

**Table 2:** Diagnostic modalities utilized in 12 cases of idiopathic facial nerve paralysis

Modality	Number of cases	Outcome (number)
No investigation done	2	Full resolution (1) Lost to follow‐up (1)
Resting UAE alone	1	Full resolution (1)
Diagnostic imaging (skull radiographs) or CT of the skull and/or brain or both, or MRI alone	1	No improvement (1)
CSF and UAE	2	Full resolution (1) Partial improvement (1)
Diagnostic imaging and UAE	2^a^	Euthanized (1^a^) Lost to follow‐up (1)
Diagnostic imaging and CSF	1	Lost to follow‐up (1)
CSF, UAE, and diagnostic imaging	3	Partial improvement (2) Full resolution (1)

a One of these cases was considered “true idiopathic” as there was no history or evidence of trauma and necropsy identified no inciting cause for facial nerve pathology. The owners elected for euthanasia because of the perceived negative effect on racing performance. Histopathological analysis of the left facial nerve identified axon dropout and nerve sheath atrophy. Histology of the spinal cord, meninges, and brain was unremarkable.

Abbreviations: CSF, cerebrospinal fluid analysis; CT, computed tomography; MRI, magnetic resonance imaging; UAE, upper airway examination.

### 3.6 Treatments

Anti‐inflammatory medications were used in 64% (41/64) of cases. Medications included flunixin meglumine (21), dexamethasone (11), phenylbutazone (9), prednisolone (3), and dimethyl sulfoxide (DMSO; 3). Antibiotics were used in 48% (31/64) of cases; antibiotic drugs included trimethoprim‐sulfonamide (17), penicillin (8), minocycline (7), gentamicin (4), enrofloxacin (2), oxytetracycline (2), and metronidazole (1). Forty‐one percent (26/64) of equids received some form of targeted ophthalmological treatment. Of the 34 animals that were unable to blink, 26 (76%) received ophthalmological treatment. Forty‐one percent (14/34) of animals underwent unilateral or bilateral temporary or permanent partial tarsorrhaphy and 1 horse had conjunctival grafting performed. Medical ophthalmic treatments are listed in Data S1. Targeted neurological drugs were administered to 31% (20/64) animals. These drugs included ponazuril (15), sulfadiazine and pyrimethamine (12), minocycline (7), phenobarbital (2), gabapentin (2), botulism antitoxin (1), West Nile antibody plasma (1), and mannitol (1). Hemodynamic support was used in 17% (11/64) animals; this support was provided as IV fluid treatment (10) or blood transfusion (1). Sixteen percent (10/64) of animals had surgery, including laceration repair and arthroscopy for suspected septic arthritis (3), ceratohyoidectomy (3), temporary tracheostomy (1), exploratory celiotomy (1), prosthetic laryngoplasty (1), and tooth extraction (1). Miscellaneous treatments included vitamin E (2), butorphanol (2), gastroprotectants (1), diphenhydramine (1), cetirizine (1), and pergolide (1). No systemic treatment was provided to 19% (12/64) of the equids.

### 3.7 Outcome

The median number of days hospitalized was 3 (IQR, 1‐6.25 days). Follow‐up was available for 86% (55/64) of the cases. Fifty‐three percent (29/55) of cases had complete resolution of FNP, 11% (6/55) had partial improvement, and 11% (6/55) had FNP that was unchanged or worse. Twenty‐fixe percent (14/55) of affected animals ultimately were euthanized (1 horse was euthanized specifically because of the FNP). Reasons for euthanasia included progression of generalized neurological signs (8), progressive weight loss (3), colic (1), and severe THO (1). Of the 8 animals euthanized for progression of neurological signs, 3 were diagnosed with EPM and 3 with neuroborreliosis, and FNP was considered a complication of their primary neurological disease. The remaining 2 animals had progressive neurological signs (seizures, recumbency) but FNP was considered to be secondary to trauma caused by their neurological disease.

### 3.8 Associations with diagnosis

Complete results of the univariable logistic regression for significant associations between clinical variables and diagnosis are presented in Table 3. Nonsignificant associations are provided in Data S1. True idiopathic and “not investigated” idiopathic cases were combined for the purpose of statistical analyses.

**Table 3:** Univariable analysis results for significant associations (*P* < .05) between clinical variables and diagnosis in 64 equids with facial nerve paralysis

Diagnosis (n)	Clinical variable	OR	95% CI	*P* value
EPM (10)	Presence of additional neurological signs			
	Yes	6.81	1.12‐41.26	.04
	No	Ref.	‐	‐
	Presence of ataxia			
	Yes	10.45	2.25‐48.66	.003
	No	Ref.	‐	‐
	Presence of a head tilt			
	Yes	6.33	1.55‐25.96	.01
	No	Ref.	‐	‐
	MMS of ataxia			
	0	Ref.	‐	‐
	4	35	1.34‐911.28	.03
	Negative for EPM intrathecal antibody production using a serum: CSF ratio			
	Yes	.02	0.0‐0.23	.002
	No	Ref.	‐	‐
	Treatment with targeted neurological drugs			
	Yes	89	4.82‐1642.99	.003
	No	Ref.	‐	‐
	Full resolution of FNP			
	Yes	5.3	1.17‐24.0	.03
	No	Ref.	‐	‐
Neuroborreliosis (5)	Presence of ataxia			
	Yes	29	1.52‐554.07	.03
	No	Ref.	‐	‐
	Negative neuroborreliosis diagnosis on CSF and serum			
	Yes	0.02	0.0‐0.47	.02
	No	Ref.	‐	‐
	Euthanized			
	Yes	14.14	1.98‐100.83	.01
	No	Ref.	‐	‐
Idiopathic FNP (12)	Presenting with a corneal ulcer			
	Yes	7.44	1.27‐43.47	.03
	No	Ref.	‐	‐
	Presenting complaint			
	FNP	Ref.	‐	‐
	Trauma	0.04	0.0‐0.84	.04
	Negative for EPM intrathecal antibody production using a serum: CSF ratio			
	Yes	21	1.01‐438.23	.05
	No	Ref.	‐	‐
	FNP partially improved			
	Yes	5.21	1.01‐26.76	.05
	No	Ref.	‐	‐
CNS disease (16)	Presenting complaint			
	FNP	Ref.	‐	‐
	Other neurological problem	13.36	2.49‐71.66	.002
	History of trauma			
	Yes	0.05	0.0‐0.97	.05
	No	Ref.	‐	‐
	Presence of ear droop			
	Yes	0.2	0.06‐0.68	.01
	No	Ref.	‐	‐
	Presence of additional neurological signs			
	Yes	14.37	2.45‐84.29	.003
	No	Ref.	‐	‐
	Presence of ataxia			
	Yes	32.09	6.8‐151.39	<.005
	No	Ref.	‐	‐
	MMS of ataxia			
	0	Ref.	‐	‐
	2	7.86	1.17‐52.95	.03
	3	15	1.51‐148.59	.02
	4	35	1.34‐911.28	.03
	Negative for EPM intrathecal antibody production using a serum: CSF ratio			
	Yes	0.12	0.02‐0.89	.04
	No	Ref.	‐	‐
	Final diagnosis of trauma			
	Yes	0.04	0.0‐0.74	.03
	No	Ref.	‐	‐
	Treatment with targeted neurological drugs			
	Yes	21.34	5.21‐87.38	<.005
	No	Ref.	‐	
	Euthanized			
	Yes	4.37	1.27‐15	.02
	No	Ref.	‐	‐
Trauma (20)	Presenting complaint			
	FNP	Ref.	‐	‐
	Trauma	119.22	11.20‐1269.35	<.005
	Generalized weakness/malaise	10.09	1.26‐80.66	.03
	Leucocytosis			
	Yes	6.41	1.3‐31.45	.02
	No	Ref.	‐	‐
	Neutrophilia			
	Yes	6.03	1.4‐25.9	.02
	No	Ref.	‐	‐
	High creatinine kinase			
	Yes	11.07	1.38‐88.92	.02
	No	Ref.	‐	‐
	History of trauma			
	Yes	47.91	9.63‐238.28	<.005
	No	Ref.	‐	‐
	Presence of muzzle deviation			
	Yes	0.3	0.1‐0.91	.03
	No	Ref.	‐	‐
	Presence of additional neurological signs			
	Yes	0.32	0.11‐0.95	.04
	No	Ref.	‐	‐
	Presence of ataxia			
	Yes	0.92	0.02‐0.54	.008
	No	Ref.	‐	‐
	Final diagnosis of CNS disease			
	Yes	0.04	0.0‐0.74	.03
	No	Ref.	‐	‐
THO (10)	Breed			
	TB	Ref.	‐	‐
	Quarter Horse	8.14	1.47‐45.23	.02
	Pony	40.71	1.61‐1032.27	.03
	Presence of ear droop			
	Yes	6.81	1.12‐41.26	.04
	No	Ref.	‐	‐
	Unable to blink on affected side			
	Yes	7.08	1.17‐42.95	.03
	No	Ref.	‐	‐
	3 or more signs of FNP present			
	Yes	7.33	1.21‐44.4	.03
	No	Ref.	‐	‐
	Presence of ocular pathology			
	Yes	17.69	2.86‐109.56	.002
	No	Ref.	‐	‐
	No abnormalities detected on diagnostic imaging of the skull			
	Yes	0.04	0.0‐0.82	.04
	No	Ref.	‐	‐
	Normal UAE			
	Yes	0.01	0.0‐0.24	.004
	No	Ref.	‐	‐
	Treatment with antibiotics			
	Yes	4.56	1.01‐20.59	.05
	No	Ref.	‐	‐
	Performance of surgery			
	Yes	4.38	1.05‐18.28	.04
	No	Ref.	‐	‐
	Performance of unilateral or bilateral temporary or permanent partial tarsorrhaphy			
	Yes	7.9	1.94‐32.26	.004
	No	Ref.	‐	‐
	Treatment with ophthalmological drugs			
	Yes	4.23	1.06‐16.92	.04
	No	Ref.	‐	‐

*Note:* The data should be interpreted thus, for example, a horse with FNP and additional neurological signs was 6.81 times more likely to have diagnosis of EPM than those without additional neurological signs (OR, 6.81; 95% CI, 1.12‐41.26; *P* = .04).

Abbreviations: CI, confidence interval; EPM, equine protozoal myeloencephalitis; FNP; facial nerve paralysis; OR, odds ratio; Ref., Reference; TB, thoroughbred; UAE, upper airway examination.

#### 3.8.1 Trauma

Animals with muzzle deviation were less likely to have a final diagnosis of trauma than those without this clinical finding (odds ratio [OR], 0.3; 95% confidence interval [CI], 0.1‐0.91; *P* = .03). Presence of additional neurological signs (eg, abnormal mentation, head tilts) and presence of ataxia were negatively associated with a diagnosis of trauma (OR, 0.32; 95% CI, 0.11‐0.95; *P* = .04 and OR, 0.92; 95% CI, 0.02‐0.54; *P* = .01, respectively).

#### 3.8.2 Central nervous system diseases

A negative association between presence of ear droop and CNS disease was found (OR, 0.20; 95% CI, 0.06‐0.68; *P* = .01). Positive associations were found between presence of additional neurological signs (OR, 14.37; 95% CI, 2.45‐84.29; *P* = .01) and presence of ataxia (OR, 32.09; 95% CI, 6.80‐151.39; *P* < .01) with CNS disease. Positive associations were found between animals that had EPM and a head tilt (OR, 6.33; 95% CI, 1.55‐25.96; *P* = .01), and MMS of 4 (OR, 35; 95% CI, 1.34‐911.28; *P* = .03).

#### 3.8.3 Idiopathic FNP

Equids presenting with a corneal ulcer were 7.44 times more likely to have a final diagnosis of idiopathic FNP than those without a corneal ulcer (95% CI, 1.27‐43.47; *P* = .03).

#### 3.8.4 Temporohyoid Osteoarthropathy (THO)

Quarter Horses were 8.14 times more likely and Pony breeds were 40.71 times more likely to have a diagnosis of THO than Thoroughbreds (95% CI, 1.47‐45.23; *P* = .02 and 95% CI, 1.61‐1032.27; *P* = .03, respectively). Positive associations were found between an ear droop (OR, 6.81; 95% CI, 1.12‐41.26; *P* = .04) and inability to blink on the affected side (OR, 7.08; 95% CI, 1.17‐42.95; *P* = .03) with THO. Equids with ≥3 signs of FNP were 7.33 times more likely to have THO than those with <3 signs of FNP (95% CI, 1.21‐44.40; *P* = .03). In addition, presence of ocular pathology was positively associated with a diagnosis of THO (OR, 17.69; 95% CI, 2.86‐109.56; *P* = .002). Equids with normal diagnostic imaging results were less likely to have THO than those with abnormal results (OR, 0.04; 95% CI, 0.0‐0.82; *P* = .04).

### 3.9 Associations with outcome

The complete results of the univariable logistic regression for significant associations between clinical variables and outcome are presented in Table 4. Nonsignificant associations are provided in Data S1.

**Table 4:** Univariable analysis results for significant associations (*P* < .05) between clinical variables and outcome in 64 equids with facial nerve paralysis

Outcome (n)	Clinical variable	OR	95% CI	*P* value
Full resolution of FNP at follow‐up (29)	Age (years)	0.89	0.83‐0.97	.01
	Presenting complaint			
	FNP	Ref.	‐	‐
	Trauma	5.03	1.21‐20.91	.03
	Chronicity of FNP			
	Acute	7	2.01‐24.32	.01
	Chronic	Ref.	‐	‐
	Presence of ptosis			
	Yes	0.35	0.13‐0.95	.04
	No	Ref.	‐	‐
	Diagnosis of EPM			
	Yes	5.3	1.17‐24	.03
	No	Ref.	‐	‐
	Treatment with targeted neurological drugs			
	Yes	3.12	1.06‐9.15	0.04
	No	Ref.	‐	‐
Euthanized (14)	Presenting complaint			
	FNP	Ref.	‐	‐
	Generalized weakness/malaise	8.56	1.39‐52.74	.02
	Abnormal mentation at presentation			
	Yes	4.8	1.21‐18.91	.03
	No	Ref.	‐	‐
	Presence of additional neurological signs			
	Yes	5.85	1.35‐25.33	.02
	No	Ref.	‐	‐
	Presence of ataxia			
	Yes	8.01	2.22‐28.96	.001
	No	Ref.	‐	‐
	MMS			
	0	Ref.	‐	‐
	2	29	1.30‐648.44	.03
	3	40.6	1.47‐1050.23	.03
	4	48.33	1.50‐1554.28	.03
	Performance of CSF analysis			
	Yes	5.32	1.56‐18.18	.01
	No	Ref.	‐	‐
	Diagnosis of neuroborreliosis			
	Yes	14.14	1.98‐100.83	0.01
	No	Ref.	‐	‐
	Diagnosis of CNS disease			
	Yes	4.37	1.27‐15	0.02
	No	Ref.	‐	‐
Partial improvement of FNP (6)	Breed			
	TB	Ref.	‐	‐
	Pony	20.33	1.24‐332.52	.04
	Rectal temperature (°F)	0.31	0.1‐0.95	.04
	GGT (U/L)	1.07	1‐1.13	.03
	Diagnosis of idiopathic FNP			
	Yes	5.21	1.01‐26.76	.05
	No	Ref.	‐	‐
	Performance of surgery			
	Yes	5.94	1.14‐30.94	.03
	No	Ref.	‐	‐
	Use of ophthalmic antifungal medication			
	Yes	12.11	2.07‐70.83	.01
	No	Ref.	‐	‐
No improvement or worsening FNP (6)	Presenting with a corneal ulcer			
	Yes	8.81	1.34‐58.08	.02
	No	Ref.	‐	‐
	Basophil count (× 10^3^/μL)	200.51	1.08‐37 213.51	.05
	Use of ophthalmic antibiotic medication			
	Yes	9.44	1.42‐62.69	.02
	No	Ref.	‐	‐

*Note:* The data should be interpreted thus, for example, horses that had a presenting complaint of trauma were 5.03 times more likely to have full resolution of the FNP than those presenting for FNP (OR, 5.03; 95% CI, 1.21‐20.91; *P* = .03).

Abbreviations: CI, confidence interval; CNS, central nervous system; CSF, cerebrospinal fluid; EPM, equine protozoal myeloencephalitis; FNP; facial nerve paralysis; GGT, gamma‐glutamyl transferase; MMS, modified Mayhew score; OR, odds ratio; Ref., Reference; TB, thoroughbred, UAE, upper airway examination.

#### 3.9.1 Full resolution

Advanced age was associated with being less likely to have full resolution of FNP (OR, 0.89; 95% CI, 0.83‐0.97; *P* = .01). Equids with an acute (within 7 days of onset) presentation were more likely to have full resolution of FNP (OR, 7; 95% CI, 2.01‐24.32; *P* = .002) than those presenting for trauma (OR, 5.03; 95% CI, 1.21‐20.91; *P* = .03) and those with a final diagnosis of EPM (OR, 5.3; 95% CI, 1.17‐24.00; *P* = .031). A positive association also was found between treatment using targeted neurological drugs and full resolution of FNP (OR, 3.12; 95% CI, 1.06‐9.15; *P* = .04). Equids presenting with ptosis were significantly less likely to have full resolution of FNP (OR, 0.35; 95% CI, 0.13‐0.95; *P* = .04).

#### 3.9.2 Partial improvement of FNP

A final diagnosis of idiopathic was positively associated with partial improvement of FNP (OR, 5.21; 95% CI, 1.01‐26.76; *P* < .05).

#### 3.9.3 FNP unchanged or worse

Equids that presented with a corneal ulcer were 8.81 times more likely to have unchanged or worse FNP reported at follow up (95% CI, 1.34‐58.08; *P* = .02).

#### 3.9.4 Euthanasia

Nonsurvival was associated with presentation for generalized weakness or malaise (OR, 8.56; 95% CI, 1.39‐52.74; *P* = .02) or abnormal mentation (OR, 4.8; 95% CI, 1.21‐18.92; *P* = .03). Nonsurvival also was associated with presence of ataxia (OR, 8.01; 95% CI, 2.22‐28.96; *P* = .001), presence of additional neurological clinical signs (OR, 5.85; 95% CI, 1.35‐25.33; *P* = .02), performance of CSF collection (OR, 5.32; 95% CI, 1.56‐18.18; *P* = .01) and diagnosis of neuroborreliosis (OR, 14.14; 95% CI, 1.98‐100.83; *P* = .01).

## 4 DISCUSSION

In this report, we describe 64 equids that were presented to our hospital with FNP. Most cases (92%) were unilateral, with left and right sides almost equally represented. A wide range of ages and breeds was affected with a nearly even distribution of sexes. A wide variety of presenting complaints ranging in severity was recorded, including several animals with debilitating illness. Thirteen animals were euthanized for a reason other than FNP; 6 of these had FNP as a clinical sign of their CNS disease. For the surviving animals, there was a good chance of complete resolution or improvement of the FNP (85% of cases).

The facial nerve has several important functions, including innervation of the muscles of facial expression, the pinna, eyelid, lip, and digastricus muscle; parasympathetic control of the salivary glands, lacrimal glands, and nasal cavity; sensation to the internal surface of the pinna and sensation of taste to the rostral 67% of the tongue. Perhaps most crucial is innervation of the eyelid and parasympathetic control of the lacrimal glands, which combine to protect the eye from keratitis sicca and exposure keratopathy. In our study, 53% of animals were unable to blink, resulting in the presence of a corneal ulcer in over 33% of cases with FNP. The need to protect the eye was well recognized, with nearly 80% of the animals that were unable to blink being provided targeted ophthalmological treatment and 41% undergoing temporary or permanent tarsorrhaphy.

The most common cause of FNP was trauma. The facial nerve exits the skull through the stylomastoid foramen and splits into the auricular, auriculopalpebral, buccal, cervical, and digastricus branches.^15^ The branches run through the superficial thin SC tissues of the face and across prominent bony landmarks of the skull (such as the supraorbital ridge), making the nerve branches susceptible to damage from even minor trauma.^16^ A diagnosis of trauma usually can be easily deduced from history, clinical signs, and hematological evidence of stress or muscle damage (increased creatine kinase activity). Our results indicate that equids with muzzle deviation were less likely to have a diagnosis of trauma; this finding likely means that the patients in the study population tended to traumatize the auricular and auriculopalpebral branches rather than the buccal branch of the nerve. We did not include cases of postanesthetic FNP in our report because most postanesthetic cases at our facility are transient and mild, and often not well represented in the medical record database, making accurate analysis of these cases problematic. Even if we had been able to include these cases, it is likely that the majority of the traumatic cases would have presented with lip droop or muzzle deviation alone. These signs result when the head of the anesthetized horse is positioned in such a way that the halter or other firm object compresses the buccal branches of the nerve as they cross the masseter muscle.^16^

A diagnosis of trauma was not associated with any of the outcome categories, presumably because of the range in severity of injury to the nerve in this category. Mild trauma might only cause a temporary loss in nerve conduction (neuropraxia), whereas more severe injury can result in complete denervation (neurotmesis).^17^ It is unlikely that the clinician's initial physical examination could determine the extent of the facial nerve damage. Therefore, we recommend against trying to prognosticate return to function until reexamination.

The second most common cause of FNP was CNS disease. Central nervous causes of FNP described in the veterinary literature include EPM, neuroborreliosis, EHV‐1 myeloencephalopathy, viral encephalitis, infectious meningitis, intracranial abscess or neoplasia, and polyneuritis equi.^12^, ^18^, ^19^, ^20^ Some CNS diseases, such as EPM and neuroborreliosis, have the potential to cause FNP without any additional neurological signs, and definitive diagnosis can be challenging.^12^, ^18^ Therefore, some of the uninvestigated idiopathic cases in our study truly may be cases of CNS disease, particularly those without CSF analysis. In addition, none of these animals were tested for *Neospora hughesi*. Although EPM caused by *N. hughesi* is much more rare than that caused by *S. neurona*, it still could cause FNP and should be considered as a differential diagnosis in these cases.^21^ At our facility, EPM is diagnosed using *S. neurona* SAG 2, 4/3 ELISA serum : CSF titer ratios, because current evidence suggests that this testing method has the greatest accuracy of the available commercial tests, with a reported accuracy of 97%.^11^, ^22^ Conversely, a confident diagnosis of Lyme disease or neuroborreliosis is much more difficult and requires confirmation at necropsy. Several horses in our study had both CSF and serum tested for antibodies against *B. burgdorferi*. Our hospital uses the multiplex (a bead‐based multiple antigen ELISA assay) which detects antibody production to outer surface proteins (Osps) *of B. burgdorferi*.^13^ Without an ante‐mortem gold standard of diagnosis, this assay has been difficult to validate and a recent study found that paired serum and CSF multiplex results could not accurately diagnose neuroborreliosis in horses with a diagnosis confirmed on necropsy examination.^23^, ^24^ For both EPM and neuroborreliosis, antemortem diagnosis is best achieved using a combination of immunodiagnostic testing, CSF cytology, clinical signs, and appropriate diagnostic testing for other neurological disorders.^13^, ^22^ In our study, univariable logistic regression analysis found positive associations between both diseases and ataxia. In addition, horses with EPM were more likely to have a head tilt. Presence of additional neurological clinical signs in equids with FNP should increase the clinician's suspicion for CNS disease and prompt CSF analysis.

Outcome of horses with FNP was variable. A diagnosis of EPM was associated with full resolution of FNP, a diagnosis of neuroborreliosis was associated with euthanasia, and the prognosis for FNP for other causes was less well defined. Some improvement was seen in idiopathic cases and no clear prognosis could be established for cases of THO. More research with a larger number of cases is needed to allow prognostication. Based on our findings, hematological and biochemistry findings are unlikely to provide information concerning the etiology of the FNP or provide any specific prognostic information for the FNP. Instead, these diagnostic tests should be used to help identify any comorbidities requiring clinical intervention.

In our study, 19% of cases were given a final diagnosis of idiopathic, but only 6% of our cases ultimately were considered “truly” idiopathic. This finding is in stark contrast to the small animal veterinary literature, in which most cases of FNP are found to be idiopathic.^3^ In human medicine, idiopathic FNP (Bell's palsy) accounts for approximately 70% of cases of FNP.^25^, ^26^ Idiopathic FNP in horses is anecdotally described as being common. However, this assessment probably represents an overestimation, and likely reflects a lack of thorough diagnostic investigation to rule out possible causes. We defined “true idiopathic” FNP as having no history or clinical signs of trauma, normal CSF analysis, unremarkable guttural pouch endoscopy, and normal diagnostic imaging of the skull. These criteria would reasonably rule out the main differential diagnoses. An additional case also was considered true idiopathic because histopathological changes in the facial nerve (axon dropout, nerve sheath atrophy), with no obvious inciting cause, were the only findings on necropsy examination. Ultimately, only 4 cases (6%) over a 19‐year time period met our inclusion criteria, suggesting that idiopathic FNP is less common than previously thought. However, selection bias is a potential limitation of our study because most cases presented to a referral hospital likely have severe or persistent FNP, and mild idiopathic cases may not be referred.

For cases in which a specific causative disease was identified, treatment was specific for the disease process. For example, animals with EPM generally were treated with ponazuril, sulfadiazine and pyrimethamine, or both. Animals that were treated with targeted neurological drugs such as these were 3.12 times more likely to have full resolution of their FNP. This finding suggests that if the FNP is caused by CNS disease, and if that disease responds to treatment (as did 60‐70% of EPM cases), the FNP also will resolve.^18^, ^27^, ^28^

These equids also received other treatments for their clinical signs. For example, 64% of animals were treated with anti‐inflammatory drugs. Because of the retrospective nature of our study, it is unknown if these medications were given with the aim of treating the FNP or if they were prescribed to manage comorbidities. In many cases, nerve dysfunction likely was associated with neuronal inflammation. In humans, Bell's palsy is commonly treated using corticosteroids. In 2007, a double‐blinded, placebo‐controlled, randomized study with 551 participants reported significant improvement in patients treated with prednisolone within 72 hours of onset.^29^ In small animals, the benefit of corticosteroid administration is not known.^30^ We did not find any association between anti‐inflammatory drug use and outcome. However, our study was limited by a large number of confounding factors, including a wide range in severity of illness. Further prospective studies, examining the use of anti‐inflammatory drugs in cases of FNP, are needed to help guide clinical decision‐making.

In conclusion, the most common causes of FNP in equids differ markedly from those in other species. The most common cause of FNP in our patient population was trauma, whereas in the human and small animal medical literature the final diagnosis often is “idiopathic.” Central nervous system disease was the second most common cause, highlighting the importance of careful consideration of the entire clinical picture when examining an equid with FNP. In our study, CNS‐affected equids often had other neurological signs, in addition to FNP. Attempting to give a prognosis for a neuropathy that has a wide range of causes of variable severity is challenging. However, for surviving equids, the prognosis for FNP generally was good.

## CONFLICT OF INTEREST DECLARATION

Authors declare no conflict of interest.

## OFF‐LABEL ANTIMICROBIAL DECLARATION

Authors declare no off‐label use of antimicrobials.

## INSTITUTIONAL ANIMAL CARE AND USE COMMITTEE (IACUC) OR OTHER APPROVAL DECLARATION

Authors declare no IACUC or other approval was needed.

## HUMAN ETHICS APPROVAL DECLARATION

Authors declare human ethics approval was not needed for this study.

## Supporting information


**Data S1**. Complete list of the variables analyzed
**Table S1**. Presenting complaint in 64 equids with facial nerve paralysis
**Table S2**. Hematology and biochemistry findings in equids (n = 42) presenting with facial nerve paralysis
**Table S3**. Cerebrospinal fluid analysis (n = 19) findings in equids presenting with facial nerve paralysis
**Table S4**. Univariable analysis results for nonsignificant associations (*P* > .05‐.2) between clinical variables and diagnosis in 64 equids with facial nerve paralysis
**Table S5**. Univariable analysis results for nonsignificant associations (*P* > .05‐.2) between clinical variables and outcome in 64 equids with facial nerve paralysis
