# Supplementary material for: Facial nerve paralysis in 64 equids: Clinical variables, diagnosis, and outcome
Source: J Vet Intern Med. 2020 Apr 6;34(3):1308–20. doi: 10.1111/jvim.15767 (PMC7255662; doi:10.1111/jvim.15767)
Supplement: Supplementary file 1 — Data S1. Complete list of the variables analyzed Table S1. Presenting complaint in 64 equids with facial nerve paralysis Table S2. Hematology and biochemistry findings in equids (n = 42) presenting with facial nerve paralysis Table S3. Cerebrospinal fluid analysis (n = 19) findings in equids presenting with facial nerve paralysis Table S4. Univariable analysis results for nonsignificant associations (P > .05‐.2) between clinical variables and diagnosis in 64 equids with facial nerve paralysis Table S5. Univariable analysis results for nonsignificant associations (P > .05‐.2) between clinical variables and outcome in 64 equids with facial nerve paralysis [file JVIM-34-1308-s001.pdf]

Supplementary Table 1 - Presenting complaint in 64 Equids with Facial Nerve Paralysis

Presenting complaint	Category
Facial nerve paralysis (18)<br>Respiratory Distress due to bilateral nasal collapse (1)	Facial Nerve Paralysis (19)
Unspecified trauma (6)<br>Head trauma (3)<br>Caught in wire fence (2)<br>Ran into wall (1)<br>Persistent neck scoliosis (1)<br>Facial swelling (1)<br>Thrashing due to drug reaction (1)	Trauma (15)
Head tilt (2)<br>Unable to open mouth (1)<br>Horner's Syndrome (1)<br>Dull (1)<br>Ataxic (8)<br>Muzzle, ear, eye, chest fasciculations (1)<br>Seizures (1)	Other Neurological Problem (15)
Weakness (2)<br>Recumbency (2)<br>Weight loss (2)<br>Decreased appetite/inappetance (3)	Generalized Weakness/Malaise (9)
Poor performance (2)<br>Left laryngeal hemiplegia/"roaring" (1)<br>Foot abscess (1)<br>Ear pruritis (2)	Other (6)

Supplementary Table 2 – Hematology and biochemistry findings in equids (n=42) presenting with facial nerve paralysis

Parameter (unit)	Normal range[9]	Median (range)	Number	Number (%) with increased results	Number (%) with decreased results
Packed Cell Volume (%)	30-47	37 (13-46)	42	0 (0%)	3 (7%)
Total solids (g/dL)	6.4-7.4	6.8 (5.4-8.4)	42	6 (14%)	8 (19%)
White Blood Cells ($x10^3/\mu L$)	5.00-12.00	7.87 (4.21-17.55)	40	8 (20%)	1 (2%)
Red Blood Cells ($x10^6/\mu L$)	5.30-10.50	8.00 (1.01-10.96)	36	2 (6%)	2 (6%)
Platelet count ($x10^3/\mu L$)	90-360	130 (69-300)	29	0 (0%)	2 (7%)
Neutrophil count ($x10^3/\mu L$)	2.18-6.96	5.84 (2.8-13.52)	40	0 (0%)	13 (33%)
Lymphocyte count ($x10^3/\mu L$)	1.32-5.86	1.89 (.62-8.01)	40	1 (3%)	12 (30%)
Monocyte count ($x10^3/\mu L$)	.05-.92	.23 (.06-.72)	37	0 (0%)	0 (0%)
Eosinophil count ($x10^3/\mu L$)	.01-1.00	.07 (.00-.69)	32	0 (0%)	3 (9%)
Basophil count ($x10^3/\mu L$)	.00-.12	.02 (.01-.72)	28	1 (4%)	N/A
Creatinine (mg/dL)	.6-1.8	1.41 (1.0-2.0)	36	3 (8%)	0 (0%)
Lactate (mmol/L)	<2.5	1.4 (.6-3.0)	9	1 (11%)	N/A
Glucose (mg/dL)	75-125	123.9 (94.6-178)	28	13 (46%)	0 (0%)
Sodium (mmol/L)	134-144	135 (125-140)	26	0 (0%)	7 (27%)
Potassium (mmol/L)	2.7-4.9	3.74 (3.06-4.93)	24	1 (4%)	0 (0%)
Chloride (mmol/L)	94-102	99 (96-103)	24	3 (13%)	0 (0%)
Carbon Dioxide (mmol/L)	24-31	28.2 (19-35)	24	5 (21%)	3 (13%)
Calcium (mg/dL)	10.7-13.4	11.75 (9.79-13.5)	24	5 (21%)	1 (4%)
Phosphorus (mg/dL)	1.9-5.4	3.07 (1.82-6.86)	16	1 (6%)	1 (6%)
Albumin (g/dL)	2.50-4.20	3.00 (2.45-3.54)	24	0 (0%)	1 (4%)
Aspartate Aminotransferase (U/L)	205-555	387 (176-1719)	25	5 (20%)	2 (8%)
Gamma-Glutamyltransferase (U/L)	12-45	39.5 (21-118)	26	5 (19%)	0 (0%)
Creatinine Kinase (U/L)	90-270	156 (62-887)	22	10 (45%)	3 (14%)
Total Bilirubin (mg/dL)	.1-1.9	3.65 (.8-10.2)	18	12 (67%)	0 (0%)
Fibrinogen (mg/dL)	150-375	399.5 (198-681)	42	24 (57%)	0 (0%)

Supplementary Table 3 – Cerebrospinal fluid analysis (n=19) findings in equids presenting with facial nerve paralysis

Parameter (unit)	Normal range[29]	Median (range)	Number (%) with elevated results	Number (%) with decreased results
White blood cells (/µL)	0-6	2 (0-73)	2 (11%)	N/A
Red blood cells (/µL)	N/A	11 (0-5200)	N/A	N/A
Total Protein (mg/dL)	5-100	87 (17-211)	6 (32%)	0 (0%)

The clinical variables analyzed in the statistical analysis: breed, sex, age, presenting complaint category, presence of a corneal ulcer, duration of FNP in days, acute/chronic, presenting temperature, presence of a fever at presentation, presenting pulse, presence of tachycardia at presentation, presenting respiratory rate, PCV, TS, presence of high total solids, presence of low total solids, white blood cell count, presence of leukocytosis, red blood cell count, platelet count, neutrophil count, presence of neutrophilia, lymphocyte count, presence of lymphopenia, monocyte count, eosinophil count, basophil count, creatinine level, glucose level, presence of hyperglycemia, sodium level, presence of hyponatremia, potassium level, chloride level, TCO2 level, presence of high TCO2, calcium level, presence of low calcium, phosphorus level, albumin level, AST level, GGT level, presence of high GGT, creatinine kinase level, presence of high creatinine kinase, total bilirubin level, presence of high total bilirubin, fibrinogen level, presence of high fibrinogen, history of trauma, right or left sided FNP, uni or bilateral FNP, presence of ptosis, presence of an ear droop, presence of muzzle deviation, presence of inability to blink, presence of lip droop, presence of 3 or more signs of FNP, presence of abnormal mentation, presence of head tilt, presence of other neurological signs, presence of eye pathology, presence of comorbidities, presence of ataxia, Modified Mayhew Score, normal upper airway examination result, performance of skull CT, performance of skull radiographs, normal skull imaging result, CSF total protein, CSF white blood cell count, CSF red blood cell count, EPM serum titer, EPM CSF titer, negative EPM result, Lyme serum OspA, Lyme serum OspC, Lyme serum OspF, Lyme CSF OspA, Lyme CSF OspC, Lyme serum OspF, negative Lyme result, final diagnosis category, days hospitalized, treatment with anti-inflammatories, treatment with antibiotics, treatment with targeted neurological drugs, performance of surgery, provision of hemodynamic support, performance of uni- or bilateral temporary or permanent partial tarsorrhaphy, treatment with eye antibiotic, treatment with eye antifungal, treatment with ophthalmological drugs, case outcome.

Supplementary Table 4 – Univariable analysis results for non-significant associations (P>0.05-0.2) between clinical variables and diagnosis in 64 equids with facial nerve paralysis
*Please note – if a parameter is not listed in the table below or in the body of the manuscript (Table 3), the P value was >0.2.

Diagnosis (n)	Clinical Variable	Odds Ratio	95% Confidence Interval	P Value
EPM (10)	Presenting complaint category<br>FNP<br>Other neurologic problems	 ref<br>4.79	 -<br>.91-25.16	 -<br>.064
	Chronicity of FNP<br>Acute<br>Chronic	 4.38<br>ref	 .70-27.30<br>-	 .113<br>-
	History of trauma<br>Yes<br>No	 .10<br>ref	 .01-1.84<br>-	 .122<br>-
	Presence of ear droop<br>Yes<br>No	 .32<br>ref	 .08-1.28<br>-	 .108<br>-
	Presence of altered mentation at presentation<br>Yes<br>No	 2.96<br>ref	 .67-13.04<br>-	 .152<br>-
	No abnormalities detected on diagnostic imaging of the skull<br>Yes<br>No	 12.6<br>ref	 .62-255.76<br>-	 .099<br>-
	Final diagnosis of Trauma<br>Yes<br>No	 .08<br>ref	 .00-1.44<br>-	 .087<br>-
	Days hospitalized	1.06	.99-1.13	.091
	Performance of uni- or bilateral temporary or permanent partial tarsorrhaphy<br>Yes<br>No	 .13<br>ref	 .01-2.42<br>-	 .173<br>-
Neuroborreliosis (5)	Sex<br>Mare<br>Castrated male	 ref<br>13.69	 -<br>.72-259.93	 -<br>.081
	Presenting complaint category<br>FNP<br>Other neurological problem<br>Generalized weakness/malaise	 ref<br>10.92<br>13.00	 -<br>.52-229.91<br>.56-303.67	 -<br>.124<br>.111
	Duration of FNP (days)	1.01	1.00-1.02	.056
	Chronicity of FNP<br>Acute<br>Chronic	 .06<br>ref	 .00-1.11<br>-	 .058<br>-
	Lymphopenia<br>Yes<br>No	 13.57<br>ref	 .60-306.64<br>-	 .101<br>-
	High TCO2			

	Yes<br>No	13.00<br>ref	.45-374.54<br>-	.135<br>-
	Presence of ear droop<br>Yes<br>No	 .06<br>ref	 .00-1.19<br>-	 .065<br>-
	Unable to blink on affected side<br>Yes<br>No	 .25<br>ref	 .04-1.73<br>-	 .161<br>-
	3 or more signs of FNP present<br>Yes<br>No	 .07<br>ref	 .00-1.27<br>-	 .072<br>-
	Presence of neurological signs (not FNP)<br>Yes<br>No	 10.64<br>ref	 .56-201.04<br>-	 .115<br>-
	Presence of ocular pathology<br>Yes<br>No	 .14<br>ref	 .01-2.68<br>-	 .192<br>-
	Modified Mayhew Score<br>0<br>1<br>2<br>3	 ref<br>9.67<br>18.45<br>9.67	 -<br>.33-281.32<br>.81-418.56<br>.33-281.32	 -<br>.187<br>.067<br>.187
	Normal Upper Airway Endoscopy<br>Yes<br>No	 10.33<br>ref	 .53-202.81<br>-	 .124<br>-
	EPM SAG 2,3,4, ELISA serum titer ratio	1.00	1.00-1.00	.062
	Treatment with anti-inflammatories<br>Yes<br>No	 .16<br>ref	 .02-1.10<br>-	 .063<br>-
	Treatment with ophthalmological drugs<br>Yes<br>No	 .12<br>ref	 .01-2.33<br>-	 .162<br>-
Idiopathic FNP (12)	Presenting complaint<br>FNP<br>Other neurologic problem<br>Generalized weakness/malaise	 ref<br>.25<br>.07	 -<br>.05-1.26<br>.00-1.40	 -<br>.093<br>.082
	Neutrophilia<br>Yes<br>No	 .10<br>ref	 .01-1.92<br>-	 .127<br>-
	High bilirubin<br>Yes<br>No	 .05<br>ref	 .00-1.35<br>-	 .075<br>-
	History of trauma<br>Yes<br>No	 .29<br>ref	 .05-1.74<br>-	 .175<br>-
	Presence of lip droop<br>Yes	 3.10	 .70-13.65	 .135

	No	ref	-	-
	Normal Upper Airway Endoscopy<br>Yes<br>No	 5.00<br>ref	 .75-33.37<br>-	 .097<br>-
	Performance of skull CT<br>Yes<br>No	 3.37<br>ref	 .58-19.46<br>-	 .175<br>-
	No abnormalities detected on diagnostic imaging of the skull<br>Yes<br>No	 6.81<br>ref	 .98-47.23<br>-	 .052<br>-
	Negative neuroborreliosis diagnosis on CSF and serum<br>Yes<br>No	 11.00<br>ref	 .48-250.87<br>-	 .133<br>-
	Final diagnosis of Trauma<br>Yes<br>No	 .06<br>ref	 .00-1.13<br>-	 .061<br>-
	Treatment with anti-inflammatories<br>Yes<br>No	 .33<br>ref	 .10-1.15<br>-	 .082<br>-
	Treatment with hemodynamic support<br>Yes<br>No	 .14<br>ref	 .01-2.63<br>-	 .191<br>-
	Use of ophthalmic antifungal medication<br>Yes<br>No	 3.97<br>ref	 .83-18.90<br>-	 .083<br>-
CNS Disease (16)	Presenting complaint<br>FNP<br>Generalized weakness/malaise	 ref<br>3.77	 -<br>.59-24.12	 -<br>.161
	Leucocytosis<br>Yes<br>No	 .13<br>ref	 .01-2.40<br>-	 .168<br>-
	Hyponatremia<br>Yes<br>No	 .26<br>ref	 .04-1.85<br>-	 .176<br>-
	High creatinine kinase<br>Yes<br>No	 .29<br>ref	 .05-1.75<br>-	 .178<br>-
	Fibrinogen (mg/dL)	.99	.99-1.00	.079
	Increased fibrinogen<br>Yes<br>No	 .38<br>ref	 .10-1.51<br>-	 .171<br>-
	FNP laterality<br>Right<br>Left	 2.37<br>ref	 .73-7.76<br>-	 .152<br>-
	3 or more signs of FNP present<br>Yes	 .45	 .14-1.38	 .161

	No	ref	-	-
	Change in mentation<br>Yes<br>No	 3.78<br>ref	 .98-14.57<br>-	 .053<br>-
	Presence of head tilt<br>Yes<br>No	 2.65<br>ref	 .74-9.51<br>-	 .136<br>-
	Presence of ocular pathology<br>Yes<br>No	 .36<br>ref	 .10-1.33<br>-	 .126<br>-
	Normal Upper Airway Endoscopy<br>Yes<br>No	 6.09<br>ref	 .92-40.18<br>-	 .061<br>-
	No abnormalities detected on diagnostic imaging of the skull<br>Yes<br>No	 21.67<br>ref	 1.10-425.06<br>-	 .053<br>-
	Negative neuroborreliosis diagnosis on CSF and serum<br>Yes<br>No	 .06<br>ref	 .00-1.45<br>-	 .084<br>-
	Final diagnosis of idiopathic<br>Yes<br>No	 .09<br>ref	 .00-1.59<br>-	 .100<br>-
	Final diagnosis of THO<br>Yes<br>No	 .11<br>ref	 .01-2.01<br>-	 .137<br>-
	Performance of surgery<br>Yes<br>No	 .10<br>ref	 .01-1.78<br>-	 .116<br>-
	Performance of uni- or bilateral temporary or permanent partial tarsorrhaphy<br>Yes<br>No	 .07<br>ref	 .00-1.28<br>-	 .073<br>-
	Full resolution of FNP<br>Yes<br>No	 2.44<br>ref	 .79-7.59<br>-	 .122<br>-
Trauma (20)	Presence of tachycardia<br>Yes<br>No	 2.90<br>ref	 .84-9.98<br>-	 .091<br>-
	High total solids<br>Yes<br>No	 3.35<br>ref	 .63-17.77<br>-	 .155<br>-
	Hyponatremia<br>Yes<br>No	 6.06<br>ref	 .99-37.09<br>-	 .051<br>-
	High TCO2<br>Yes<br>No	 4.82<br>ref	 .70-33.32<br>-	 .111<br>-
	High AST			

	Yes<br>No	5.13<br>ref	.75-35.33<br>-	.096<br>-
	Fibrinogen (mg/dL)	1.00	1.00-1.01	.175
	High fibrinogen<br>Yes<br>No	 3.09<br>ref	 .75-12.70<br>-	 .118<br>-
	Change in mentation<br>Yes<br>No	 .29<br>ref	 .05-1.75<br>-	 .177<br>-
	Final diagnosis of EPM<br>Yes<br>No	 .08<br>ref	 .00-1.44<br>-	 .087<br>-
	Final diagnosis of idiopathic<br>Yes<br>No	 .06<br>ref	 .00-1.13<br>-	 .061<br>-
	Final diagnosis of THO<br>Yes<br>No	 .08<br>ref	 .00-1.44<br>-	 .087<br>-
	Treatment with hemodynamic support<br>Yes<br>No	 3.22<br>ref	 .89-11.63<br>-	 .075<br>-
THO (10)	Presenting with a corneal ulcer<br>Yes<br>No	 4.33<br>ref	 .73-25.61<br>-	 .106<br>-
	Chronicity of FNP<br>Acute<br>Chronic	 .26<br>ref	 .06-1.11<br>-	 .070<br>-
	Low total solids<br>Yes<br>No	 3.41<br>ref	 .68-17.25<br>-	 .137<br>-
	Hyponatremia<br>Yes<br>No	 5.61<br>ref	 .60-52.50<br>-	 .131<br>-
	High creatinine kinase<br>Yes<br>No	 .13<br>ref	 .01-2.84<br>-	 .194<br>-
	Presence of muzzle deviation<br>Yes<br>No	 3.48<br>ref	 .57-21.22<br>-	 .177<br>-
	Final diagnosis of CNS disease<br>Yes<br>No	 .11<br>ref	 .01-2.01<br>-	 .137<br>-
	Final diagnosis of trauma<br>Yes<br>No	 .08<br>ref	 .00-1.44<br>-	 .087<br>-
	Treatment with anti-inflammatories<br>Yes<br>No	 4.38<br>ref	 .72-26.63<br>-	 .108<br>-
	Treatment with targeted			

	neurological drugs<br>Yes<br>No	 .08<br>ref	 .00-1.44<br>-	 .087<br>-
	Treatment with ophthalmological antibiotics<br>Yes<br>No	 3.68<br>ref	 .97-14.03<br>-	 .056<br>-
	Full resolution of FNP<br>Yes<br>No	 .29<br>ref	 .07-1.33<br>-	 .111<br>-
	Partial resolution of FNP<br>Yes<br>No	 3.30<br>ref	 .60-18.22<br>-	 .171<br>-
	No improvement or worsening FNP<br>Yes<br>No	 3.30<br>ref	 .60-18.22<br>-	 .171<br>-

Supplementary Table 5 – Univariable analysis results for non-significant associations (P>0.05-0.2) between clinical variables and outcome in 64 equids with facial nerve paralysis
*Please note – if a parameter is not listed in the table below or in the body of the manuscript (Table 4), the P value was >0.2.

Diagnosis (n)	Clinical Variable	Odds Ratio	95% Confidence Interval	P Value
Full resolution of FNP at follow up (29)	Sex<br>Mare<br>Intact male	 ref<br>3.89	 -<br>.54-28.15	 -<br>.179
	Presenting complaint<br>FNP<br>Other neurologic problem	 ref<br>2.99	 -<br>.75-11.98	 -<br>.122
	Rectal temperature (°F)	1.91	.94-3.89	.075
	Total solids (g/dL)	.49	.18-1.33	.160
	Red blood cells (/µL)	1.45	.91-2.31	.121
	Neutrophil count ($x10^3$/µL)	1.17	.93-1.47	.181
	Neutrophilia<br>Yes<br>No	 3.03<br>ref	 .78-11.69<br>-	 .108<br>-
	Potassium (mmol/L)	.19	.02-1.65	.133
	Presence of ear droop<br>Yes<br>No	 .38<br>ref	 .14-1.03<br>-	 .058<br>-
	Unable to blink on affected side<br>Yes<br>No	 .40<br>ref	 .15-1.08<br>-	 .071<br>-
	No abnormalities detected on diagnostic imaging of the skull<br>Yes<br>No	 2.88<br>ref	 .72-11.52<br>-	 .135<br>-
	Final diagnosis of CNS disease<br>Yes<br>No	 2.44<br>ref	 .79-7.59<br>-	 .122<br>-
	Final diagnosis of THO<br>Yes<br>No	 .29<br>ref	 .07-1.33<br>-	 .111<br>-
Died/euthanized (14)	Age (years)	1.06	.98-1.15	.161
	Breed<br>TB<br>Quarter Horse	 ref<br>.12	 -<br>.01-2.29	 -<br>.159
	Sex<br>Mare<br>Intact male	 ref<br>2.73	 -<br>.77-9.63	 -<br>.119
	Presenting complaint category<br>FNP<br>Other neurological problem	 ref<br>4.79	 -<br>.91-25.16	 -<br>.064
	Chronicity of FNP<br>Acute<br>Chronic	 .29<br>ref	 .08-1.07<br>-	 .064<br>-

	Tachycardia<br>Yes<br>No	 2.35<br>ref	 .67-8.21<br>-	 .179<br>-
	Total solids (g/dL)	2.45	.73-8.16	.145
	High total solids<br>Yes<br>No	 4.69<br>ref	 .85-25.85<br>-	 .076<br>-
	Platelet count (x10^3/μL)	0.97	.94-1.01	.155
	Lymphopenia<br>Yes<br>No	 2.88<br>ref	 .63-13.20<br>-	 .173<br>-
	Carbon Dioxide (mmol/L)	.86	.68-1.08	.192
	Total Bilirubin (mg/dL)	1.40	.88-2.23	.155
	High Bilirubin<br>Yes<br>No	 9.53<br>ref	 .44-207.37<br>-	 .151<br>-
	FNP laterality<br>Right<br>Left	 .38<br>ref	 .11-1.35<br>-	 .136<br>-
	Presence of ptosis<br>Yes<br>No	 2.28<br>ref	 .65-7.96<br>-	 .195<br>-
	Modified Mayhew Score<br>0<br>1	 ref<br>9.67	 -<br>.33-281.32	 -<br>.187
	Treatment with antibiotics<br>Yes<br>No	 .37<br>ref	 .11-1.26<br>-	 .111<br>-
	Performance of uni- or bilateral temporary or permanent partial tarsorrhaphy<br>Yes<br>No	 .31<br>ref	 .05-1.86<br>-	 .200<br>-
Partial improvement of FNP (6)	Breed<br>TB<br>Draft<br>Arabian	 ref<br>12.20<br>12.20	 -<br>.90-165.03<br>.90-165.03	 <br>.060<br>.060
	Presenting complaint category<br>FNP<br>Other neurological problem<br>Trauma<br>Generalized weakness/malaise	 ref<br>.09<br>.27<br>.14	 -<br>.00-1.68<br>.04-1.91<br>.01-2.81	 -<br>.105<br>.191<br>.198
	Heart rate (beats per minute)	.93	.84-1.03	.144
	Phosphorus (mg/dL)	1.98	.74-5.29	.172
	High GGT<br>Yes<br>No	 14.33<br>ref	 .50-411.82<br>-	 .120<br>-
	Presence of ptosis<br>Yes<br>No	 3.80<br>ref	 .58-24.81<br>-	 .164<br>-

	Presence of ear droop<br>Yes<br>No	 3.43<br>ref	 .52-22.37<br>-	 .198<br>-
	Unable to blink on affected side<br>Yes<br>No	 3.54<br>ref	 .54-23.15<br>-	 .187<br>-
	3 or more signs of FNP present<br>Yes<br>No	 3.67<br>ref	 .56-23.94<br>-	 .175<br>-
	Presence of ocular pathology<br>Yes<br>No	 3.65<br>ref	 .71-18.77<br>-	 .122<br>-
	Negative for EPM intrathecal antibody production using a serum:CSF ratio<br>Yes<br>No	 7.74<br>ref	 .35-170.10<br>-	 .194<br>-
	Final diagnosis of THO<br>Yes<br>No	 3.30<br>ref	 .60-18.22<br>-	 .171<br>-
	Performance of uni- or bilateral temporary or permanent partial tarsorrhaphy<br>Yes<br>No	 4.13<br>ref	 .82-20.78<br>-	 .085<br>-
No improvement or worsening FNP (6)	Breed<br>TB<br>Pony<br>Donkey	 ref<br>8.14<br>24.43	 -<br>.65-101.28<br>.83-723.00	 -<br>.103<br>.064
	Rectal temperature (°F)	.40	.12-1.34	.136
	Respiratory rate (breaths per minute)	1.05	.98-1.12	.170
	Platelet count (x10^3/μL)	1.02	1.00-1.04	.073
	Neutrophil count (x10^3/μL)	.39	.12-1.40	.147
	Monocyte count (x10^3/μL)	106.71	.35-32250.75	.109
	Presence of ear droop<br>Yes<br>No	 3.43<br>ref	 .52-22.37<br>-	 .198<br>-
	Presence of muzzle deviation<br>Yes<br>No	 6.92<br>ref	 .37-129.11<br>-	 .195<br>-
	Unable to blink on affected side<br>Yes<br>No	 3.54<br>ref	 .54-23.15<br>-	 .187<br>-
	3 or more signs of FNP present<br>Yes<br>No	 3.67<br>ref	 .56-23.94<br>-	 .175<br>-
	Normal Upper Airway Endoscopy<br>Yes	 .13	 .01-2.82	 .191

	No	ref	-	-
	No abnormalities detected on diagnostic imaging of the skull<br>Yes<br>No	 <br>.09<br>ref	 <br>.00-1.84<br>-	 <br>.118<br>-
	Final diagnosis of THO<br>Yes<br>No	 3.30<br>ref	 .60-18.22<br>-	 .171<br>-
	Treatment with targeted neurological drugs<br>Yes<br>No	 <br>.14<br>ref	 <br>.01-2.69<br>-	 <br>.195<br>-
	Performance of uni- or bilateral temporary or permanent partial tarsorrhaphy<br>Yes<br>No	 <br><br>4.13<br>ref	 <br><br>.82-20.78<br>-	 <br><br>.085<br>-
